# Implementation of Exome Sequencing in Prenatal Diagnosis and Impact on Genetic Counseling: The Polish Experience

**DOI:** 10.3390/genes13050724

**Published:** 2022-04-21

**Authors:** Anna Kucińska-Chahwan, Maciej Geremek, Tomasz Roszkowski, Julia Bijok, Diana Massalska, Michał Ciebiera, Hildeberto Correia, Iris Pereira-Caetano, Ana Barreta, Ewa Obersztyn, Anna Kutkowska-Kaźmierczak, Paweł Własienko, Małgorzata Krajewska-Walasek, Piotr Węgrzyn, Lech Dudarewicz, Waldemar Krzeszowski, Magda Rybak-Krzyszkowska, Beata Nowakowska

**Affiliations:** 1 Department of Medical Genetics, Institute of Mother and Child, 01-211 Warsaw, Poland; maciej.geremek@imid.med.pl (M.G.); ewa.obersztyn@imid.med.pl (E.O.); anna.kutkowska@imid.med.pl (A.K.-K.); pawel.wlasienko@imid.med.pl (P.W.); beata.nowakowska@imid.med.pl (B.N.); 2 Department of Obstetrics and Gynecology, Institute of Mother and Child, 01-211 Warsaw, Poland; tomcior1@gmail.com; 3 Department of Gynecology Oncology and Obstetrics, Center of Postgraduate Medical Education, 01-809 Warsaw, Poland; julia.bijok@gmail.com (J.B.); diana_massalska@wp.pl (D.M.); 4 Second Department of Obstetrics and Gynecology, Center of Postgraduate Medical Education, 01-809 Warsaw, Poland; michal.ciebiera@gmail.com; 5 Cytogenetics Unit, Department of Human Genetics, Nacional Health Institute Doutor Ricardo Jorge, 1649-016 Lisbon, Portugal; hildeberto.correia@jcs.pt; 6 Molecular Genetics Unit, Department of Human Genetics, National Health Institute Doutor Ricardo Jorge, 1649-016 Lisbon, Portugal; iris.caetano@jcs.pt; 7 Medical Genetics Service, Joaquim Chaves Saúde, 1495-148 Oeiras, Portugal; ana.barreta@jcs.pt; 8 Department of Genetics and Clinical Immunology, National Institute of Tuberculosis and Lung Diseases, 01-138 Warsaw, Poland; malgorzata.krajewskawalasek@gmail.com; 9 Department of Obstetrics, Perinatology and Gynaecology, Medical University of Warsaw, 02-097 Warsaw, Poland; piotr.wegrzyn@wum.edu.pl; 10 Department of Genetics, Polish Mother’s Memorial Hospital-Research Institute, 93-338 Lodz, Poland; lechdudarewicz@gmail.com; 11 Department of Perinatology and Gynecology, Polish Mother’s Memorial Hospital Research Institute, 93-338 Lodz, Poland; waldemar.krzeszowski@gmail.com; 12 Salve Medica, 91-211 Lodz, Poland; 13 Department of Obstetrics and Perinatology, University Hospital in Krakow, 31-501 Warsaw, Poland; rybaczka@interia.pl; 14 Hi-Gen Medical Centre, 30-552 Krakow, Poland

**Keywords:** fetal anomalies, prenatal diagnosis, ultrasound, exome sequencing, genomic variant, genotype–phenotype correlation

## Abstract

Background: Despite advances in routine prenatal cytogenetic testing, most anomalous fetuses remain without a genetic diagnosis. Exome sequencing (ES) is a molecular technique that identifies sequence variants across protein-coding regions and is now increasingly used in clinical practice. Fetal phenotypes differ from postnatal and, therefore, prenatal ES interpretation requires a large amount of data deriving from prenatal testing. The aim of our study was to present initial results of the implementation of ES to prenatal diagnosis in Polish patients and to discuss its possible clinical impact on genetic counseling. Methods: In this study we performed a retrospective review of all fetal samples referred to our laboratory for ES from cooperating centers between January 2017 and June 2021. Results: During the study period 122 fetuses were subjected to ES at our institution. There were 52 abnormal ES results: 31 in the group of fetuses with a single organ system anomaly and 21 in the group of fetuses with multisystem anomalies. The difference between groups was not statistically significant. There were 57 different pathogenic or likely pathogenic variants reported in 33 different genes. The most common were missense variants. In 17 cases the molecular diagnosis had an actual clinical impact on subsequent pregnancies or other family members. Conclusions: Exome sequencing increases the detection rate in fetuses with structural anomalies and improves genetic counseling for both the affected couple and their relatives.

## 1. Introduction

Congenital anomalies, as defined by the World Health Organization (WHO), are structural or functional abnormalities that occur during the intrauterine life. They can be identified prenatally, at birth or any time after birth and occur in approximately 2–3% of live births and 20% of spontaneously aborted fetuses [1,2]. As the underlying etiology of congenital anomalies includes genetic and environmental factors, current guidelines recommend chromosomal microarray analysis (CMA) as a first-tier prenatal test for fetal anomalies [3]. Prenatal karyotyping can detect clinically relevant chromosomal aberrations in about 32% of fetuses with structural anomalies and CMA increases the diagnostic yield by at least 6% [4,5]. However, most of the anomalous fetuses remain without a genetic diagnosis. Exome sequencing (ES) is a powerful tool to identify sequence variants across the protein-coding regions and is increasingly used in postnatal patients. ES is also a promising method to detect the underlying genetic etiology in the fetuses with structural anomalies and normal results of routine testing [6]. The main advantage of this approach is the possibility of identification of pathogenic variants across the exome and not narrowed to only the selected genes. ES is a phenotype-driven test and interpretation of detected variants is performed with the use of existing molecular and clinical data. The American College of Medical Genetics and Genomics (ACMG) criteria for classifying sequence variants are correlated with a clinical presentation [7]. A detected variant can be classified as pathogenic/likely pathogenic or as benign/likely benign if a particular combination of evidence of pathogenicity or benign impact is present. The remaining variants not consistent with the abovementioned criteria are classified as variants of unknown significance (VUS). In this context, a precise distinction of different fetal phenotypes with the use of Human Phenotype Ontology (HPO) terminology is essential for establishing both the diagnosis in the clinical case and general indications for prenatal ES [8,9]. Each term in the HPO describes a phenotypic abnormality and can be used to classify the anomalies in a standardized manner. The semantic clarity is necessary for communication between clinicians and scientists and for integration of biomedical human data and model organism data. There are bioinformatics tools using HPO terms (e.g., Exomiser—a JAVA program) that prioritize variants according to user-defined criteria and find potential disease-causing variants [10]. However, differences between fetal and postnatal phenotypes and limited access to additional diagnostic tests in a fetus make the interpretation of prenatal ES challenging. Therefore, a large amount of data available in public repositories and scientific reports is required to implement ES in routine prenatal diagnosis. The aim of this paper is to present the results of the first 122 prenatal ES analyses performed in our genetic department and to discuss its possible clinical impact on genetic counseling.

## 2. Materials and Methods

### 2.1. General Information

The Polish Society of Obstetricians and Gynecologists recommends at least four sonographic evaluations during pregnancy, which is in line with the international guidelines [11]. Patients at risk for fetal abnormality (age at delivery ≥35 years, family history of genetic or structural anomalies or abnormal ultrasound findings in current pregnancy) are offered genetic counseling and testing in terms of the National Prenatal Screening Program (NPSP) [12]. The Human Genetics Department at the Mother and Child Institute is certified by the Cytogenetic External Quality Assessment Service (CEQAS) and Polish Society of Human Genetics (pol. PTGC) and performs genetic tests for over a dozen obstetric gynecology centers that provide prenatal care from all country regions. Routine prenatal genetic testing performed at our department comprises CMA supplemented by karyotyping based on a single flask cell culture.

We performed a retrospective analysis of the results of ES conducted at our laboratory between January 2017 and June 2021. Written informed consent for molecular testing was obtained from all individuals after appropriate genetic counseling. Clinical and family history was taken in all cases prior to testing. Participants were informed that fetal ES would be performed in terms of research; thus, only results relevant to fetal anomalies would be reported back to parents and incidental findings from the ACMG recommended list would not be evaluated nor reported [13]. All individuals consented to the use of their de-identified data for research purposes. The study design was approved by an internal bioethics committee. Indications for fetal exome testing, molecular type, reporting status and clinical significance of detected variants, mode of inheritance, genotype–phenotype correlation, pregnancy outcome and impact of the molecular diagnosis on post-test genetic counselling were analyzed. Each fetal anomaly was labeled according to HPO terminology with HP identifiers [8,9]. We arranged fetal phenotypes into 9 categories: as anomalies of the central nervous system (HP:0002011), face (HP:000271), cardiovascular system (HP:0001626), abdomen (HP:0001438), genitourinary system (HP:0000119), musculoskeletal system (HP:0033127), neural tube defects (HP:0045005), non-immune hydrops fetalis (HP:0001790) and multisystem anomalies (i.e., fetuses with anomalies of two or more categories).

### 2.2. Exome Sequencing Procedure

In all cases, prior to exome sequencing, a chromosomal microarray was performed using the oligonucleotide array platform CytoSure Constitutional v3 (8 × 60 k) (Oxford Gene Technology, Oxford, UK) with approximately 60.000 probes across the genome. Data were analyzed with the CytoSure Interpret Software (OGT) which provided an average resolution of 120 kb. Subsequently, DNA specimens isolated from fetal samples were sent to the external laboratory for sequencing (CeGaT, Tübingen, Germany). The sequencing procedure was performed on the NovaSeq6000 (Illumina, San Diego, CA, USA) using SureSelect Human All Exon v.6 (Agilent, Santa Clara, CA, USA) for exome capture and library preparation. The resulting raw sequence data (FASTQ format files) were post-processed on site. Short reads were mapped against the human genome reference sequence (GRCh38/hg38) using the Burrows–Wheeler Alignment (BWA) and stored as Binary Sequence Alignment Map (BAM) files. Following alignment, we used the Genome Analysis Toolkit (GATK) software for variant calling, ANNOVAR for variant annotation and the CoNIFER algorithm for CNV detection from the exome sequencing data.

### 2.3. Exome Variant Interpretation

Filtering of variants was conducted as a semi-rigid strategy and not precluded to reconsider any of the disregarded variants. Initial filters looked for quality control and an allele frequency, either from the open database (gnomAD < 0.01) or in-house database (<0.05; approximately 1000 postnatal samples sequenced in the same platform and processed using the same pipeline). Subsequent filtration steps were based on the molecular impact of the variant, known gene–disease correlation considering allele zygosity and clinical significance based on variant databases (ClinVar, HGMD), in silico evaluation of pathogenicity (SIFT, Polyphen-2, MutationTaster, CADD) and review of the literature. The filtering strategy was supported by the Exomiser tool in parallel to manual searching [10]. Selected variants were classified in accordance with the ACMG classification system and judged if apparently relevant to an observed fetal phenotype [7]. In each case, interpretation of the variant was discussed in a panel group of at least four participants (clinical geneticist, fetal medicine specialist, laboratory scientist and bioinformatician). Expected pathogenic or likely pathogenic variants (ACMG class 5 or 4, respectively) were verified by Sanger sequencing of the fetal DNA. Parental origin was tracked by Sanger sequencing of DNA isolated from peripheral blood of both parents. Variants of unknown significance (VUS; ACMG class 3) were verified, tracked and reported if they were found in trans (on the other allele) with the pathogenic or likely pathogenic variant in an autosomal recessive condition that fit the fetal phenotype. After completion of the analysis, we obtained pathogenic or likely pathogenic variants that explained fetal phenotype; these variants were classified as diagnostic variants or abnormal ES results, and thus, they are referred to further in this paper. Subsequently, in cases with normal ES results we proceeded to the expanded analysis. In this step, we obtained variants in disease-related genes that potentially could explain observed fetal anomalies but did not achieve scores for pathogenic/likely pathogenic variants or variants with a strong in silico prediction of deleteriousness in genes with a limited level of evidence for disease association (also referred to as candidate genes). These variants are referred to VUS thereinafter. The turnaround time of ES was around 12 weeks in each case, excluding time for the tissue culture and Sanger variants verification.

### 2.4. Outcome and Clinical Impact

ES results and post-test counsel were conveyed directly to patients by clinical geneticists from our center or to the referring clinical geneticist. Information on outcome was collected and divided into three categories: termination of pregnancy (TOP), livebirth and stillbirth. The impact of molecular diagnosis was subdivided into two categories: expected impact, defined as genetic risk assessment, and actual clinical impact, defined as altered reproductive management or molecular diagnosis in other family members.

### 2.5. Statistics

Statistical analysis was performed using STATA 12 (StataCorp LP, College Station, TX, USA). Means, medians and percentages were used to present descriptive statistics. A chi-square test was used to assess the differences between categorical data. A *p*-value < 0.05 was considered statistically significant.

## 3. Results

### 3.1. Cohort Characteristics

During the study period, 122 fetuses were subjected to ES at our institution. The mean maternal and gestational age at diagnosis was 31 years (range: 21–41 years) and 19.5 weeks (range: 12–35 weeks), respectively. Family history was positive in seven cases (similarly affected previous fetus, an affected parent or consanguineous parents) but no molecular diagnosis had previously been given. No exposure to any recognized teratogens or harmful environmental factors was identified in any of the patients. A detailed ultrasound examination and an invasive procedure was performed in each case (amniocentesis 114/122; 93.5%, chorionic villus sampling 6/122; 4.9%, fetal blood sampling 2/122; 1.6%). The first 11 (9.0%) samples were analyzed as trio exomes (fetus–mother–father), whereas the next cases were analyzed as fetus-only ES with targeted Sanger sequencing for detected variants in both parents. All cases were referred due to structural anomalies or fetal hydrops. We divided all cases into two groups: fetuses with a single organ system anomaly (64/122; 52.5%) and fetuses with multisystem anomalies (58/122; 47.5%).

### 3.2. Results by Phenotype

Overall, there were 52 (42.6%) abnormal ES results: 31 in the group of fetuses with a single organ system anomaly (31/64; 48.4%) and 21 in the group of fetuses with multisystem anomalies (21/58; 36.2%) (Table 1 and Table 2). The difference between groups was not statistically significant *X*^2^ (1, *n* = 122) = 1.86, *p* = 0.17. In a univariate logistic regression, musculoskeletal anomalies increased the odds of an abnormal ES result by 2.5 fold (OR 2.5, 95% CI 1.2–5.4; *p* = 0.016) whereas cardiovascular defects decreased the odds of an abnormal ES result by 0.34 fold (OR 0.34, 95% CI 0.14–0.8; *p* = 0.014).

### 3.3. Results by Molecular Diagnosis

There were 57 different pathogenic or likely pathogenic variants reported in 33 different genes. The most common were missense variants (28/57; 49.1%), followed by frameshift variants (13/57; 22.8%), nonsense variants (12/57; 21.1%) and splicing variants (4/57; 7.0%); 34 variants were novel (34/57; 59.6%), whereas the remaining 23 were previously reported (23/57; 40.4%). The molecular mechanism of pathogenicity in the majority of novel variants was the same as previously reported for specific genes (31/34; 83.8%). The mechanism of pathogenicity in the three remaining novel variants was different than frequently reported, but the fetal phenotype was consistent with the literature data (Table 2 ID 10, 23 and 45). Variants of unknown significance were detected in 39 out of 70 fetuses without definite genetic diagnosis (55.7%; 1–5 VUS per fetus); 77 different variants were identified in 73 genes. Four genes were present twice (*COL2A1*, *EVC2*, *GLI3* and *KMT2C*, presented in Table 3 with ID 63, 91, 66, 81, 76, 83, 62 and 84, respectively). The mode of inheritance was autosomal dominant (AD) in 31 cases of abnormal results (31/52; 59.6%), autosomal recessive (AR) in 18 cases (18/52; 34.6%) and X-linked (XL) in 3 cases (3/52; 5.8%). In 24 cases, based on sonographic findings, the referring clinician suggested a specific genetic diagnosis (19.7%; 24/122), which was confirmed by ES in 15 cases (62.5%; 15/24).

### 3.4. Outcome and Clinical Impact

The outcome of the pregnancy was known in 107 cases (87.7%; 107/122): in 54 cases the pregnancy was terminated upon parental request, because of fetal major anomalies, after appropriate counseling (TOP), 47 pregnancies ended in livebirth and 6 fetuses were stillborn. (Table 2, Table 3 and Table 4). In all 52 families with abnormal fetal ES results, the genetic risk was estimated based on the molecular diagnosis causing expected impact on individuals. Actual clinical impact was observed in 17 cases (13.9%). Reproductive management was altered in 13 families either by using highly effective contraception (*n* = 2) or by altered medical management in the subsequent pregnancy (*n* = 11), such as in vitro fertilization with preimplantation genetic diagnosis and embryo selection, CVS with targeted molecular diagnosis, ultrasound and prenatal care in the reference center. In four families, molecular diagnosis was established not only in the fetus, but also in other affected relatives.

## 4. Discussion

### 4.1. Diagnostic Yield of Prenatal ES

Previous studies report a wide range of the added value of prenatal ES, ranging between 6 and 80% [14,15]. The discrepancy between various studies is most probably due to different study populations, inclusion criteria and sample size. Two large prospective studies on 610 and 234 fetuses with various sonographic anomalies, including isolated, increased NT, indicated a diagnostic yield of ES of 8.5% and 10%, respectively [16,17]. In contrast, Fu et al. reported a diagnostic yield of ES of 24% in a group of 191 fetuses with structural malformations; however, they excluded fetuses with isolated, large NT or cystic hygroma [18]. In our cohort, the added value of ES was even higher and reached 42.6%, similar to 39.4% reported by He et al. [19]. This may be due to a relatively high proportion of cases with skeletal anomalies included and can be considered as selection bias. We found a high diagnostic rate of 62,1% in fetuses with musculoskeletal system abnormalities, which is consistent with the data from other authors [16,17,18,19,20,21]. Interestingly, in our cohort cardiac defects significantly decreased the odds of an abnormal ES result. The proportion of detected pathogenic or likely pathogenic variants in fetuses with isolated cardiac defects was 16.7%, slightly higher than 11.5% reported in a large prospective cohort study and systematic review [22]. The diagnostic yield in both isolated, specific anomalies and recognizable multisystem sets of anomalies is probably more valuable for clinical practice than the overall diagnostic yield in heterogenous cohorts.

### 4.2. Interpretation of Detected Variants

The process of reaching the ES result is based on discarding variants that are unlikely to be responsible for the disease and leaving a variant or variants correlated with the phenotype. In our research, Exomiser was helpful in the detection of likely causative variants, but the final decision relied on man. The interpretation of VUS in a prenatal setting presented a particular challenge [23,24]. In three cases of limb-body wall complex (LBWC), the condition where severe anomalies are not compatible with postnatal life, the significance of variants detected in *CEP120*, *FLNB* and *COL2A1* genes was unknown (Table 3 ID 89, 90 and 91). Complexity of the disorder, lack of the specified HPO term for LBWC and absence of postnatal patients with LBWC caused problems in sequence variants interpretation. Potential risks of both overdiagnosing the variant as pathogenic and misdiagnosing as a benign finding should be considered. Therefore, in selected cases, a consensus decision made in a multidisciplinary panel hastened the diagnosis. Lefebvre et al. described a blind to phenotype, genotype-first approach that we do not consider appropriate—without knowing the phenotype, the variants cannot be interpreted [25].

### 4.3. Molecular Mechanism, Inheritance Mode and Their Clinical Impact 

The most common molecular changes in our cohort were missense variants, which is in line with other studies [16,17,18,26]. In the majority of cases, the mechanism of pathogenicity was in accordance with previous reports. In three cases, however, the mechanism was different and a detailed sonographic description enabled a correct diagnosis (Table 2 ID 10, 23 and 45). For instance, two novel heterozygous variants (missense and frameshift) in the *NEK9* gene were found in a fetus, which presented with micrognathia, akinesia, an abnormally shaped abdomen and an increased level of free β-hCG (14.1 MoM) at 12 weeks of gestation (Table 2 ID 45). Pathogenic missense variants in the *NEK9* gene have previously been reported in individuals with arthrogryposis, avascular necrosis of the femoral head and cardiac defects, which was not consistent with the anomalies detected in our case. However, a literature review revealed a report of two related families, in which five offspring were homozygous for a nonsense variant in *NEK9* exactly phenotypically matching our case [27]. To our knowledge, this is the first report of the lethal congenital contracture syndrome (OMIM # 617022) caused by the *NEK9* variant and diagnosed in the first trimester.

The mode of inheritance in our cohort was similar to that described in other studies [16,17,18,19,20,21,22,23,25]. The majority of diagnostic variants were de novo autosomal dominant, most fetuses with autosomal recessive variants were compound heterozygotes and X-linked inheritance was the least common.

By having information on a detected diagnostic variant and its inheritance trait, we were able to estimate the recurrence risk in affected couples. Furthermore, we observed that 13 families benefited from the exome results to act according to their beliefs and make conscious decisions. As noticed by several authors, the introduction of prenatal ES is important in a wide context of genetic counseling [26,28,29,30,31,32]. It can offer considerable advantages for both the couple of the affected fetus and other family members. The turnaround time in our study was around 12 weeks; thus, the results did not have a direct impact on clinical management in current pregnancies. However, by gaining experience the turnaround time can be reduced, which is essential for the decision making in ongoing pregnancies.

## 5. Conclusions

To conclude, exome sequencing increases the detection rate in fetuses with structural anomalies and improves genetic counseling. Establishing a molecular diagnosis has clinical impact on subsequent pregnancies, as well as other family members.

## Figures and Tables

**Table 1:** Diagnostic variants and variants of unknown significance detected by exome sequencing in different phenotypic groups of fetuses.

Fetal Phenotype	Diagnostic Variants		VUS	
	*n*/N	% (95% CI)	*n*/N	% (95% CI)
CNS	4/10	40.0 (16.7–68.8)	2/10	20.0 (4.6–52.1)
Face	1/1	100.0 (16.8–100.0)	0/1	0.0 (0.0–83.3)
Cardiovascular	2/12	16.7 (3.5–46.0)	7/12	58.3 (31.9–80.7)
Abdomen	0/2	0.0 (0.0–71.0)	1/2	50.0 (9.5–90.6)
Genitourinary	4/5	80.0 (36.0–98.0)	0/5	0.0 (0.0–48.9)
Musculoskeletal	18/29	62.1 (44.0–77.4)	5/29	17.2 (7.1–35.0)
NTD	0/3	0.0 (0.0–61.8)	2/3	66.7 (20.2–94.4)
NIHF	2/2	100.0 (29.0–100.0)	0/2	0.0 (0.0–71.0)
Multisystem	21/58	36.2 (25.0–49.1)	22/58	37.9 (26.5–50.8)
Total	52/122	42.6 (34.2–51.5)	39/122	32.0 (24.3–40.7)

CNS—central nervous system, NTD—neural tube defects, NIHF—nonimmune hydrops fetalis.

**Table 2:** Sonographic findings in fetuses with abnormal exome sequencing—detailed information.

ID	Gene	Disorder and Mode of Inheritance	Transcript	Coding Alteration	Variant Type	Variant Reporting Status	Zygosity	Inheritance	Fetal Phenotype	Outcome
CNS										
1	*TREX1*	# 225750 Aicardi–Goutières syndrome 1 (AR)	NM_033629.6	c.[37A > C] c.[341G > A]	missense missense	novel reported	compound heterozygous	mat pat	ventriculomegaly HP:0002119, cerebral calcifications HP:0002514, abnormal cortical gyration HP:0002536	Stillbirth
2	*CEP290*	# 610188 Joubert syndrome 5 (AR)	NM_025114.4	c.[1666delA] c.[2424C > A]	frameshift nonsense	reported novel	compound heterozygous	pat mat	ventriculomegaly HP:0002119	TOP
3	*TUBA1A*	# 611603 Lissencephaly 3 (AD)	NM_001270399.1	c.[985A > C]	missense	novel	heterozygous	de novo	agenesis of corpus callosum HP:0001274, cerebellar vermis hypoplasia HP:0001320	Livebirth
4	*PQBP1*	# 309500 Renpenning’s syndrome (XL)	NM_001032382.2	c.[459_462del]	frameshift	reported	hemizygous	mat	ventriculomegaly HP:0002119, abnormal cortical gyration HP:0002536	Stillbirth
Face										
5	*IRF6*	# 119300 Van der Woude syndrome 1 (AD)	NM_006147.3	c.[250C > T]	missense	reported	heterozygous	mat	cleft upper lip HP:0000204	Livebirth
Cardiovascular										
6	*GATA6*	# 600001 Pancreatic agenesis and congenital heart defects (AD)	NM_005257.6	c.[1477C > T]	nonsense	novel	heterozygous	mat	CAT HP:0001660	Livebirth
7	*KDM6A*	# 300867 Kabuki syndrome 2 (XL)	NM_021140.3	c.[3016C > T]	nonsense	reported	hemizygous	de novo	HLHS HP:0004383	TOP
Genitourinary										
8	*PKHD1*	# 263200 Polycystic kidney disease 4, with or without hepatic disease (AR)	NM_138694.4	c.[10489delC] c.[1774G > A]	frameshift nonsense	novel novel	compound heterozygous	mat pat	cystic kidneys HP:0000107	Livebirth
9	*PKHD1*	# 263200 Polycystic kidney disease 4, with or without hepatic disease (AR)	NM_138694.4	c.[107C > T] c.[107C > T]	missense missense	reported reported	homozygous	mat pat	cystic kidneys HP:0000107	Livebirth
10	*ETFDH*	# 231680 Multiple acyl-CoA dehydrogenase deficiency (AR)	NM_001281738.1	c.[1191C > A] c.[1560delA]	nonsense frameshift	novel novel	compound heterozygous	mat pat	cystic kidneys HP:0000107	Livebirth
11	*TMEM67*	# 613550 Nephronophthisis 11 (AR)	NM_153704.6	c.[1843T > C] c.[1843T > C]	missense missense	reported reported	homozygous	mat pat	cystic kidneys HP:0000107	Livebirth
Musculoskeletal										
12	*FGFR3*	# 187600 Thanatophoric dysplasia, type I (AD)	NM_000142.5	c.[742C > T]	missense	reported	heterozygous	de novo	short limbs HP:0009826, short ribs HP:0000773	Livebirth
13	*FGFR3*	# 187600 Thanatophoric dysplasia, type I (AD)	NM_000142.5	c.[742C > T]	missense	reported	heterozygous	de novo	short limbs HP:0009826, short ribs HP:0000773	Livebirth
14	*FGFR3*	# 187600 Thanatophoric dysplasia, type I (AD)	NM_000142.5	c.[742C > T]	missense	reported	heterozygous	de novo	short limbs HP:0009826, short ribs HP:0000773	Livebirth
15	*FGFR3*	# 187600 Thanatophoric dysplasia, type I (AD)	NM_000142.5	c.[742C > T]	missense	reported	heterozygous	de novo	short limbs HP:0009826, short ribs HP:0000773	TOP
16	*FGFR3*	# 187600 Thanatophoric dysplasia, type I (AD)	NM_000142.5	c.[742C > T]	missense	reported	heterozygous	de novo	short limbs HP:0009826, short ribs HP:0000773	TOP
17	*FGFR3*	# 187600 Thanatophoric dysplasia, type I (AD)	NM_000142.5	c.[1118A > G]	missense	reported	heterozygous	de novo	short limbs HP:0009826, short ribs HP:0000773	TOP
18	*FGFR3*	# 187601 Thanatophoric dysplasia, type II (AD)	NM_022965.4	c.[1612A > G]	missense	reported	heterozygous	de novo	cloverleaf skull HP:0002676, short ribs HP:0000773, short limbs HP:0009826	Livebirth
19	*COL1A2*	# 166210 Osteogenesis imperfecta, type II (AD)	NM_000089.4	c.[2486G > A]	missense	novel	heterozygous	pat (germinal mosaicism)	decreased skull ossification HP:0004331, short ribs HP:0000773, short limbs HP:0009826, multiple fractures HP:0005855	TOP
20	*COL1A2*	# 166210 Osteogenesis imperfecta, type II (AD)	NC_000007.13	g.[94053760G > A]	splicing	novel	heterozygous	de novo	decreased skull ossification HP:0004331, short ribs HP:0000773, short limbs HP:0009826, multiple fractures HP:0005855	TOP
21	*COL1A2*	# 166210 Osteogenesis imperfecta, type II (AD)	NM_000089.4	c.[1739G > T]	missense	novel	heterozygous	de novo	decreased skull ossification HP:0004331, short ribs HP:0000773, short limbs HP:0009826, multiple fractures HP:0005855	TOP
22	*COL1A2*	# 259420 Osteogenesis imperfecta, type III (AD)	NM_000089.4	c.[3269G > T]	missense	reported	heterozygous	de novo	short limbs HP:0009826	TOP
23	*COL2A1*	# 271700 Spondyloperipheral dysplasia (AD)	NM_001844.5	c.[4313_4314delinsAA]	nonsense	novel	heterozygous	de novo	short ribs HP:0000773, mesomelia HP:0003027	Livebirth
24	*COL2A1*	# 200610 Achondrogenesis, type II or hypochondrogenesis (AD)	NM_033150.3	c.[2815G > T]	missense	novel	heterozygous	de novo	short limbs HP:0009826, short ribs HP:0000773	TOP
25	*COL2A1*	COL2A1-related skeletal dysplasia (AD)	NM_001844.5	c.[1546G > A]	missense	reported	heterozygous	de novo	short limbs HP:0009826	N/A
26	*DYNC2H1*	# 613091 Short-rib thoracic dysplasia 3 with or without polydactyly (AR)	NM_001377.3	c.[5911C > T] c.[9044A > G]	nonsense missense	novel reported	compound heterozygous	pat mat	short limbs HP:0009826, short ribs HP:0000773	TOP
27	*DYNC2H1*	# 613091 Short-rib thoracic dysplasia 3 with or without polydactyly (AR)	NM_001377.3	c.[9010C > T] c.[9044A > G]	missense missense	novel reported	compound heterozygous	mat pat	short limbs HP:0009826	Livebirth
28	*BMP2*	# 617877 Short stature, facial dysmorphism, and skeletal anomalies with or without cardiac anomalies (AD)	NM_001200.4	c.[840_841insAACAC]	frameshift	novel	heterozygous	pat	micrognathia HP:0000347, Pierre Robin sequence HP:0000201, polyhydramnios HP:0001561 in III trimester	Livebirth
29	*NIPBL*	# 122470 Cornelia de Lange syndrome 1 (AD)	NM_133433.4	c.[3152del]	frameshift	novel	heterozygous	de novo	micrognathia HP:0000347, Pierre Robin sequence HP:0000201, absent radius HP:0003974, absent thumb HP:0009777, hand oligodactyly HP:0001180, finger syndactyly HP:0006101	TOP
Hydrops										
30	*RIT1*	# 615355 Noonan syndrome 8 (AD)	NM_006912.6	c.244T > C	missense	reported	heterozygous	de novo	non-immune hydrops fetalis HP:0001790	TOP
31	*PTPN11*	# 163950 Noonan syndrome 1 (AD)	NM_002834.5	c.[188A > G]	missense	reported	heterozygous	de novo	non-immune hydrops fetalis HP:0001790	N/A
Multisystem										
32	*PIEZO2*	# 248700 Marden–Walker syndrome (AD)	NM_022068.4	c.[8056C > T]	missense	novel	heterozygous	de novo	micrognathia HP:0000347, Dandy–Walker malformation HP:0001305, omphalocele HP:0001539, talipes HP:0001883	N/A
33	*PIEZO2*	*PIEZO2*-related phenotype (AD)	NM_022068.4	c.[140C > A]	missense	novel	heterozygous	de novo	absence of the sacrum HP:0010305, talipes HP:0001883, AVSD HP:0006695, LAI HP:0011537, interrupted inferior vena cava with azygous continuation HP:0011671	Livebirth
34	*FGFR3*	# 187600 Thanatophoric dysplasia, type I (AD)	NM_000142.5	c.[742C > T]	missense	reported	heterozygous	de novo	short limbs HP:0009826, short ribs HP:0000773, frontal bossing HP:0002007, ventriculomegaly HP:0002119	TOP
35	*EVC*	# 225500 Ellis–Van Creveld syndrome (AR)	NM_153717.2	c.[33_34del] c.[33_34del]	frameshift frameshift	novel novel	homozygous	mat pat	short limbs HP:0009826, short ribs HP:0000773, postaxial hand polydactyly HP:0001162, AVSD HP:0006695, HAA HP:0012304	Livebirth
36	*DYNC2H1*	# 613091 Short-rib thoracic dysplasia 3 with or without polydactyly (AR)	NM_001377.3	c.[4267C > T] c.[11413T > G]	missense missense	missense novel	compound heterozygous	mat pat	single ventricle heart HP:0001750, short limbs HP:0009826, talipes HP:0001883, SUA HP:0001195	TOP
37	*TRPV4*	*TRPV*-related skeletal dysplasia (severe phenotype) (AD)	NM_021625.5	c.[ 2187C > G]	missense	novel	heterozygous	de novo	absence of the sacrum HP:0010305, short ribs HP:0000773, short limbs HP:0009826, talipes HP:0001883, left atrial isomerism HP:0011537	TOP
38	*NIPBL*	# 122470 Cornelia de Lange syndrome 1 (AD)	NM_015384.5	c.[7319dupA]	nonsense	novel	heterozygous	de novo	micrognathia HP:0000347, short ribs HP:0000773, short humerus HP:0005792, bowed humerus HP:0003865, absent forearm HP:0005632, absent hand HP:0004050, diaphragmatic hernia HP:0000776, cystic kidneys HP:0000107, thickened NF HP:0000474	TOP
39	*EFTUD2*	# 610536 Mandibulofacial dysostosis, Guion-Almeida type (AD)	NM_001142605.2	c.[2593_2596del]	frameshift	novel	heterozygous	de novo	micrognathia HP:0000347, Pierre Robin sequence HP:0000201, basal ganglia cysts HP:0006799	TOP
40	*MYO18B*	# 616549 Klippel–Feil syndrome 4, autosomal recessive, with myopathy and facial dysmorphism (AR)	NM_001318245.2	c.[6436C > T] c.[6436C > T]	nonsense nonsense	novel novel	homozygous	mat pat	thoracic hemivertebrae HP:0008467, 3–4 finger syndactyly HP:0006097, positional foot deformity HP:0005656, thickened NF HP:0000474	N/A
41	*STAG2*	# 301022 Mullegama–Klein–Martinez syndrome (XL)	ENST00000218089.13	c.[318C > G]	nonsense	novel	hemizygous	de novo	CAT HP:0001660, congenital diaphragmatic hernia HP:0000776, spina bifida HP:0002414	Livebirth
42	*DHCR7*	# 270400 Smith–Lemli–Opitz syndrome (AR)	NM_001163817.2	c.[452G > A] c.[452G > A]	nonsense nonsense	reported reported	homozygous	mat pat	postaxial hand polydactyly HP:0001162, talipes bilateral HP:0001883, LAI HP:0011537, TAPVR HP:0005160	Livebirth
43	*CC2D2A*	# 612284 Meckel syndrome 6 (AR)	NM_001080522.2	c.[3289delG] c.[4804T > C]	splicing missense	reported novel	compound heterozygous	pat mat	postaxial hand polydactyly HP:0001162, postaxial foot polydactyly HP:0001830, encephalocele HP:0002084, cystic kidneys HP:0000107	TOP
44	*CEP290*	# 610188 Joubert syndrome 5 (AR)	NM_025114.4	c.[1992del] c.[5182G > T]	frameshift nonsense	reported reported	compound heterozygous	pat mat	cystic kidneys HP:0000107, aplasia of the cerebellar vermis HP:0006817	Livebirth
45	*NEK9*	# 617022 Lethal congenital contracture syndrome 10 (AR)	NM_033116.6	c.[115_116dup] c.[1615T > C]	frameshift missense	novel novel	compound heterozygous	pat mat	fetal akinesia sequence HP:0001989, abnormally shaped abdomen (protuberant abdomen) HP:0001538	TOP
46	*BICD2*	# 618291 Spinal muscular atrophy, lower extremity-predominant, 2B (AD)	NM_001003800.2	c.[2100C > A]	missense	novel	heterozygous	de novo	fetal akinesia sequence HP:0001989, choroid plexus cyst HP:0002190, hydrops fetalis HP:0001789	TOP
47	*ACTA1*	# 161800 Nemaline myopathy 3, autosomal dominant or recessive (AD)	NM_001100.4	c.[478G > A]	missense	reported	heterozygous	de novo	overlapping fingers bilateral HP:0010557, knees fixed extension bilateral HP:0005085, talipes bilateral HP:0001883, hydrops fetalis HP:0001789, polyhydramnios HP:0001561	Stillbirth
48	*GBE1*	# 232500 Glycogen storage disease IV (AR)	NC_000003.11	g.[81627070_81627073del] g.[81698005A > G]	splicing splicing	novel reported	compound heterozygous	pat mat	fetal akinesia sequence HP:0001989, antecubital pterygia bilateral HP:0009760, hydrops fetalis HP:0001789	TOP
49	*PHGDH*	# 256520 Neu–Laxova syndrome 1 (AR)	NM_006623.4	c.[1447_1462del] c.[1447_1462del]	frameshift frameshift	novel novel	homozygous	pat mat	fetal akinesia sequence HP:0001989, disproportionate short trunk HP:0003521, lissencephaly HP:0001339, cerebellar hypoplasia HP:0001321, cleft upper lip HP:0000204, microphthalmia HP:0000568	TOP
50	*MAGEL2*	# 615547 Schaaf–Yang syndrome (AD)	NM_019066.5	c.[1853del]	frameshift	novel	heterozygous	de novo	fetal akinesia sequence HP:0001989, hydrops fetalis HP:0001789	TOP
51	*AGRN*	# 615120 Myasthenic syndrome, congenital, 8, with pre- and postsynaptic defects (AR)	NM_198576.4	c.[4657delT] 1p36.33(961,395-1,109,913)x1	frameshift deletion	novel novel	compound heterozygous	mat pat	fetal akinesia sequence HP:0001989, hydrops fetalis HP:0001789	Stillbirth
52	*RIT1*	# 615355 Noonan syndrome 8 (AD)	NM_001256820.2	c.[162G > T]	missense	reported	heterozygous	de novo	VSD HP:0006695, ileus HP:0002595, cystic hygroma HP:0000476	TOP

AVSD—atrioventricular canal defect, CAT—common arterial trunk (truncus arteriosus), CNS—central nervous system, HAA—hypoplastic aortic arch, HLHS—hypoplastic left heart, LAI—left atrial isomerism, NF—nuchal fold, SUA—single umbilical artery, TAPVR—total anomalous pulmonary venous return, VSD—ventricular septal defect, N/A—not available; names of the genes are given according to HUGO Gene Nomenclature Committee (HGNC), names of the disorders are given according to Online Mendelian Inheritance in Man database (OMIM), phenotypic descriptions with HP identifiers are given according to Human Phenotype Ontology (HPO); mode of inheritance: AD-autosomal dominant, AR—autosomal recessive, XL—X-linked.

**Table 3:** Sonographic findings in fetuses with VUSs detected in exome sequencing—detailed information.

ID	Fetal Phenotype	Gene	Outcome
CNS			
53	ventriculomegaly HP:0002119	*LAMA1*	TOP
54	cerebellar vermis hypoplasia HP:0001320	*KMT2D, MED13, COG8, ARHGEF2*	N/A
Cardiovascular			
55	VSD HP:0001629	*ARID1B*	Livebirth
56	HLHS HP:0004383	*RIT1, CDK13, CEP41, TGFBR1*	TOP
57	TGA HP:0001669	*CHD3, DNAH1, ESCO2*	Livebirth
58	single ventricle heart HP:0001750, TGA HP:0001669	*ABL1, AFF4*	Livebirth
59	pulmonary valve atresia HP:0010882	*TNXB, PACS1*	Livebirth
60	pulmonary valve atresia HP:0010882	*GATAD2B*	TOP
61	mitral regurgitation HP:0001653, cardiomegaly HP:0001640	*TTN*	TOP
Abdomen			
62	heterotaxy HP:0030853	*TENT5A, KMT2C, PIEZO1*	Livebirth
Musculoskeletal			
63	short limbs HP:0009826	*COL2A1, LTBP3*	N/A
64	short limbs HP:0009826	*PDE4D, COL11A2, TWIST1*	N/A
65	short limbs HP:0009826, talipes HP:0001883	*COL10A1, TGFBR1, CLCN7*	N/A
66	femoral bowing HP:0002980, talipes HP:0001883	*LAMA5, ZNF407, PYGM, EVC2*	N/A
67	iniencephaly HP:0010674, arthrogryposis-like hand anomaly HP:0005612, talipes HP:0001883	*LGI4*	TOP
NTD			
68	spina bifida HP:0002414	*ACAN, ERCC6, SON*	TOP
69	spina bifida HP:0002414	*ACAN, PLEKHM1*	TOP
Multisystem			
70	ventriculomegaly HP:0002119, absent septum pellucidum HP:0001331, cleft upper lip HP:0000204	*NEK1, DYNC2H1*	Livebirth
71	ventriculomegaly HP:0002119, cerebellar hypoplasia HP:0001321, cerebellar vermis hypoplasia HP:0001320, Dandy–Walker malformation HP:0001305, abnormal cortical gyration HP:0002536, short ribs HP:0000773, short limbs HP:0009826	*SMARCB2*	N/A
72	ventriculomegaly HP:0002119, cerebellar hypoplasia HP:0001321, cerebellar vermis hypoplasia HP:0001320, DORV HP:0001719, VSD HP:0001629, radial club hand HP:0004059	*ANKRD11*	TOP
73	ventriculomegaly HP:0002119, congenital diaphragmatic hernia HP:0000776, pyelectasis HP:0010945, talipes HP:0001883	*CTC1, EBF4*	Livebirth
74	ventriculomegaly HP:0002119, microphthalmia HP:0000568, VSD HP:0001629	*ZDHHC9, GPC3, CNKSR2*	N/A
75	ventriculomegaly HP:0002119, microphthalmia HP:0000568, DORV HP:0001719	*MYCN, MED12L, ZFPM2, NOTCH1, EP300*	N/A
76	abnormal cortical gyration HP:0002536, microphthalmia HP:0000568, cataract HP:0000518, abnormal sex determination HP:0012244 (phenotype female, genotype male)	*GLI3*	Livebirth
77	agenesis of corpus callosum HP:0001274, cerebellar vermis hypoplasia HP:0001320, knees fixed extension HP:0005085, overlapping fingers HP:0010557	*NALCN, MAPK8IP3, DST, KIF1A*	Livebirth
78	anencephaly HP:0002323, spina bifida HP:0002414, abnormal heart morphology HP:0001627	*NCK2, BOC*	Livebirth
79	cleft upper lip HP:0000204, hypoplasia of right ventricle HP:0004762, VSD HP:0001629	*KCNH2, MEIS2*	TOP
80	CAT HP:0001660, VSD HP:0001629, preaxial foot polydactyly HP:0001841, SUA HP:0001195	*MKKS*	Livebirth
81	DORV HP:0001719, absent radius HP:0003974	*EVC2*	TOP
82	AVSD HP:0006695, hand polydactyly HP:0001161, foot polydactyly HP:0001829, unilateral renal agenesis HP: 0000122	*SMO*	N/A
83	CAT HP:0001660, short ribs HP:0000773, scoliosis HP:0002650, preaxial foot polydactyly HP:0001841, unilateral renal agenesis HP: 0000122, cystic hygroma HP:0000476	*GLI3, DLG5*	Livebirth
84	omphalocele HP:0001539, radial club hand HP:0004059, cystic hygroma HP:0000476	*KMT2C*	Livebirth
85	micrognathia HP:0000347, absent forearm HP:0005632, absent hand HP:0004050, radial club hand HP:0004059, VSD HP:0001629, diaphragmatic hernia HP:0000776	*COLEC11*	Stillbirth
86	micrognathia HP:0000347, cystic kidneys HP:0000107, cystic hygroma HP:0000476	*HESX1*	TOP
87	micrognathia HP:0000347, fetal akinesia sequence HP:0001989, non-immune hydrops fetalis HP:0001790	*ACADVL*	TOP
88	fetal akinesia sequence HP:0001989, 2–3 finger syndactyly HP:0001233	*SYNE2, COL6A1*	TOP
89	LBWC HP:N/A	*CEP120*	TOP
90	LBWC HP:N/A	*FLNB*	TOP
91	LBWC HP:N/A	*COL2A1*	TOP

AVSD—atrioventricular canal defect, CAT—common arterial trunk (truncus arteriosus), CNS—central nervous system, DORV—double outlet right ventricle, HLHS—hypoplastic left heart, LBWC—limb-body wall complex, N/A—not available, NTD—neural tube defect, SUA—single umbilical artery, TGA—transposition of great arteries, VSD—ventricular septal defect; names of the genes are given according to HUGO Gene Nomenclature Committee (HGNC), names of the disorders are given according to Online Mendelian Inheritance in Man database (OMIM), phenotypic descriptions with HP identifiers are given according to Human Phenotype Ontology (HPO).

**Table 4:** Sonographic findings in fetuses with normal ES detected in exome sequencing—detailed information.

ID	Fetal Phenotype	Outcome
CNS		
92	cerebellar vermis hypoplasia HP:0001320, ventriculomegaly HP:0002119	Livebirth
93	ventriculomegaly HP:0002119	TOP
94	absent septum pellucidum HP:0001331	Livebirth
95	ventriculomegaly HP:0002119	Livebirth
Cardiovascular		
96	HLHS HP:0004383	TOP
97	single ventricle heart HP:0001750, CAT HP:0001660	TOP
98	lymphangioma HP:0100764	Livebirth
Abdomen		
99	omphalocele HP:0001539	Livebirth
Genitourinary		
100	cystic kidneys HP:0000107	Livebirth
Musculoskeletal		
101	absent tibia HP:0009556, fibular aplasia HP:0002990, absent foot HP:0011301, radial club hand HP:0004059, hand monodactyly HP:0004058	TOP
102	aplasia of the fingers HP:0009380 in one hand, 2–3 finger syndactyly HP:0001233 and triphalangeal thumb HP:0001199 in the other hand	Livebirth
103	skull bone defect with intact skin HP:0001362, micrognathia HP:0000347, proptosis HP:0000520	Livebirth
104	short limbs HP:0009826, craniosynostosis HP:0001363	Livebirth
105	absent radius HP:0003974, hand oligodactyly HP:0001180	TOP
106	short limbs HP:0009826	N/A
NTD		
107	spina bifida HP:0002414	Livebirth
Multisystem		
108	ventriculomegaly HP:0002119, tetralogy of Fallot HP:0001636	TOP
109	ventriculomegaly HP:0002119, ileus HP:0002595, hydronephrosis HP:0000126, unilateral renal agenesis HP:0000122, PRUV HP:N/A, SUA HP:0001195	Livebirth
110	spina bifida HP:0002414, congenital diaphragmatic hernia HP:0000776	TOP
111	LBWC HP:N/A	TOP
112	encephalocele HP:0002084, left atrial isomerism HP:0011537	TOP
113	ventriculomegaly HP:0002119, hypoplasia of the cerebellum HP:0007360, tetralogy of Fallot HP:0001636	TOP
114	single ventricle heart HP:0001750, heterotaxy HP:0030853, SUA HP:0001195	TOP
115	micrognathia HP:0000347, absence of the sacrum HP:0010305, short limbs HP:0009826, arthrogryposis-like hand anomaly HP:0005612, talipes HP:0001883, foot polydactyly (8 toes) HP:0001829, ventriculomegaly HP:0002119, CAT HP:0001660, thickened NF HP:0000474	Stillbirth
116	knees fixed extension HP:0005085, talipes HP:0001883, ileus HP:0002595, ascites HP:0001791	TOP
117	fused cervical vertebrae HP:0002949, posterior fossa cyst HP:0007291	Livebirth
118	talipes HP:0001883, ileus HP:0002595	Livebirth
119	LBWC HP:N/A	TOP
120	congenital diaphragmatic hernia HP:0000776	Livebirth
121	ventriculomegaly HP:0002119, cystic kidneys HP:0000107, thickened NF HP:0000474	N/A
122	cardiomegaly HP:0001640, short limbs HP:0009826, hydrops fetalis HP:0001789, polyhydramnios HP:0001561	Livebirth

CAT—common arterial trunk (truncus arteriosus), CNS—central nervous system, HLHS—hypoplastic left heart, LBWC—limb-body wall complex, N/A—not available, NF—nuchal fold, NTD—neural tube defect, PRUV—persistent right umbilical artery, SUA—single umbilical artery; phenotypic descriptions with HP identifiers are given according to Human Phenotype Ontology (HPO).

## Data Availability

Data supporting reported results can be found in tables.
